# Miller–Payne Grading and 70-Gene Signature Are Associated With Prognosis of Hormone Receptor-Positive, Human Epidermal Growth Factor Receptor 2-Negative Early-Stage Breast Cancer After Neoadjuvant Chemotherapy

**DOI:** 10.3389/fonc.2021.735670

**Published:** 2021-09-24

**Authors:** Liye Wang, Rongzhen Luo, Qianyi Lu, Kuikui Jiang, Ruoxi Hong, Kaping Lee, Ping Zhang, Danyang Zhou, Shusen Wang, Fei Xu

**Affiliations:** Department of Medical Oncology, Sun Yat-Sen University Cancer Center, State Key Laboratory of Oncology in South China, Collaborative Innovation Center for Cancer Medicine, Guangzhou, China

**Keywords:** breast cancer, neoadjuvant chemotherapy, Miller–Payne system, gene expression signature, prognostic

## Abstract

**Introduction:**

HR+/HER2− breast cancer (BC) has a much lower pathological complete response (pCR) rate to neoadjuvant chemotherapy (NAC). Therefore, to better stratify the relapse risk for HR+/HER2− non-pCR populations, it is essential to accurate identification new prognostic markers.

**Materials and Methods:**

The study retrospectively analyzed 105 stage II–III patients who were diagnosed with HR+/HER2− BC and received NAC followed by breast and axilla surgery between 2013 and 2019 in Sun Yat-Sen University Cancer Center. The Miller–Payne (MP) grading system was used to evaluate pathological responses to NAC. The 70-gene signature was used to classify the prognosis signatures.

**Results:**

Among the 105 patients, the study demonstrated that larger tumor size and lower progesterone receptor level at baseline and larger tumor size postoperative were statistically significantly associated with worse disease-free survival (DFS) (*p* = 0.004, *p* = 0.021, and *p* = 0.001, respectively). Among 54 patients who underwent the 70-gene assays, 26 (48.1%) had a low-risk signature; 28 (51.9%) patients had a high-risk signature. Patients with poor response (MP grades 1–2) were more likely to with a high-risk 70-gene signature than those with good response (MP grades 4–5). The final analysis showed that DFS was longer in the low-risk group than in the high-risk group [52.4 vs. 36.1 months of the median DFS, hazard ratio (HR) for recurrence, 0.29; 95% confidence interval (CI), 0.10–0.80; *p* = 0.018]. DFS was longer in the good response (MP grades 3–4) group than in the poor response (MP grades 1–2) group (94.7% vs. 60% of the patients free from recurrence; HR, 0.16; 95% CI, 0.05–0.47; *p* = 0.037). When stratified by MP grades combined with the 70-gene signature, subgroup analyses showed the good-response low-risk group with the best DFS, whereas the poor-response high-risk group showed the worst DFS (*p* = 0.048). Due to the short median follow-up time of 34.5 months (5.9–75.1 months), MP grades and the 70-gene signature did not show significant prognostic value for overall survival.

**Conclusion:**

The study showed that analysis of MP grades combined with the 70-gene signature with residual NAC-resistant breast samples has a significant correlation with DFS.

## Introduction

Hormone receptor-positive (HR+)/human epidermal growth factor receptor 2-negative (HER2−) breast cancer (BC) accounts for about 50–60% of all invasive breast carcinomas (1). HR+/HER2− patients with locally advanced stages may receive neoadjuvant chemotherapy (NAC) to reduce tumor burden and potentially facilitate breast or axillary conservation (2). Previous studies have demonstrated higher rates of pathological complete response (pCR) to NAC in triple-negative and HER2+ BC, but with a much lower rate in HR+/HER2− patients (3–5). However, pCR is not a surrogate endpoint for prediction of long-term clinical benefit in HR+/HER2− patients, such as disease-free survival (DFS) and overall survival (OS) (6). In particular, for HR+/HER2− BC, various multigene expression assays have been developed and validated to have prognostic value (7, 8). Patients who have residual invasive carcinoma after the receipt of NAC for HR+/HER2− BC have poor prognoses. Therefore, to better stratify the relapse risk for HR+/HER2− non-pCR populations, it is essential to accurate identification new prognostic markers.

The 70-gene signature (MammaPrint) is a prognostic tool that classifies tumors into groups that are associated with a good prognosis or a poor prognosis based on the risk of distant metastases at 5 and at 10 years (7). MammaPrint has been approved by the Food and Drug Administration (FDA), which can distinguish patients who are at significant risk for distant metastases and death from those at low risk (9). The prognostic value of 70-gene signature has been validated in a range of studies (9, 10). Furthermore, the results showed that the 70-gene signature increased independent prognostic information to that provided by commonly used clinicopathological factors. The Miller–Payne (MP) grading system is a widely accepted and frequently used method, and it is an independent predictor of DFS or OS (11). Prognostic value of the 70-gene signature in HR+/HER2− early-stage BC after NCT is unclear. Therefore, the 70-gene assay combined with MP grading system to assess the prognosis value of HR+/HER2− early-stage BC has great clinical significance.

Integrating tumor size and nodal status with tumor grade and genomic signatures could provide accurate prognostic estimates for HR+/HER2− BC (12, 13). The risk factors for early recurrence and for late recurrence are largely the same (14, 15). There is a need for more accurate predictive markers to guide intensive adjuvant therapy in HR+/HER2− BC after NAC. However, integrating the 70-gene signature with MP grading system for HR+/HER2− early-stage BC after NAC prognostic estimates is uncertain. Here, we designed this study to examine prognosis value of the 70-gene signature combined with MP grading system in patients who underwent NAC for HR+/HER2− early-stage BC.

## Materials and Methods

### Patient Material

**Figure 1:**
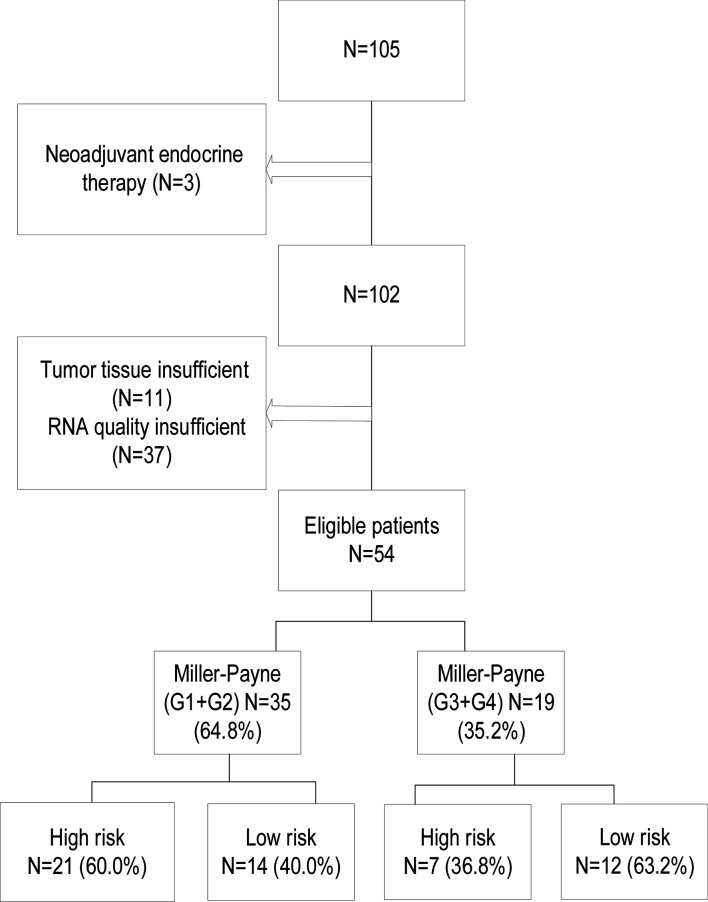
The patient selection and the classification of the 70-gene signature and Miller–Payne pathological grades.

The flowchart of the study is shown in **Figure 1**. The clinical study has been approved by the ethical committee of Sun Yat-Sen University Cancer Center (ethics approval number of clinical study project: B2020-329). Formalin-fixed paraffin-embedded tumor specimens and clinical data were retrospectively collected from a consecutive of 105 HR+/HER2− BC who received NAC between 2013 and 2019. The criteria for inclusion were as follows: (1) histologically confirmed invasive carcinoma at core needle biopsy; (2) clinical stages II–III; (3) histochemical examination of ER ≥ 10%, HER2 negative; (4) both NAC and surgical procedures performed in our hospital; (5) MP grades available after surgery; and (6) never received chemotherapy, radiation, and endocrine therapy before NAC. All patients were accepted at least two cycles of NAC. Patients with no or insufficient tumor samples as determined by a pathologist and insufficient RNA for testing were excluded, resulting in a total of 54 patients enrolled suitable for the 70-gene assay.

### Molecular and Clinical Characteristics

The 70-gene expression profile was assessed from the formalin-fixed paraffin-embedded resection breast samples blinded to clinical and pathological data. The paraffin blocks were cut into tissue sections with a thickness of 3–5 µm to make white slices without cover glass and then dewaxed to obtain enough tumor RNA for genetic testing (16, 17). RNA extraction and amplification were performed as previously described (18). Only specimens with at least 50% tumor cells were further analyzed. To assess the mRNA expression level of the 70 genes, RNA was hybridized with a standard reference to the custom-designed diagnostic chip, each containing oligonucleotide probes for the profiles in triplicate or more. Tumors were classified as high or low genomic risk as described previously (7).

The pathological response to NAC was assessed by the MP grading system (11), compared with the pathology changes in the resected tumor specimens with the tissues before chemotherapy:

Grade 1: No change or some alteration to individual malignant cells but no reduction in overall cellularity;
Grade 2: A minor loss of tumor cells but overall cellularity still high; up to 30% loss;
Grade 3: Between an estimated 30% and 90% reduction in tumor cells;
Grade 4: A marked disappearance of tumor cells such that only small clusters or widely dispersed individual cells remain; more than 90% loss of tumor cells;
Grade 5: No malignant cells identifiable in sections from the site of the tumor, only vascular fibroelastotic stroma remains often containing macrophages. However, ductal carcinoma *in situ* (DCIS) may be present.

pCR was defined as no histological evidence of malignancies or only *in situ* residuals in breast tissue after surgery and complete disappearance of lymph node metastasis.

Grades 1 and 2 are categorized as poor response, and Grades 3–5 are categorized as good response.

### Following Up

The follow-up data including the dates of recurrence and death were collected through the outpatient service, telephone, and the way to the hospital review, until death or the date of the last follow-up (on May 1, 2021). The median follow-up time was 37.5 months (2.4–92.3 months). DFS was calculated from the date of surgery to the date of disease relapse (local or distant relapse or death from any cause). OS was calculated from the date of surgery to the date of death or the latest follow-up.

### Statistical Analysis

Analyses were performed using SPSS version 26. The differences in patients and tumor characteristics between the 70-gene high- and low-risk signature were tested using Fisher’s exact test. Logistic regression was applied to identify variables predictive of MP grades. The results of the model were expressed as odds ratio (OR) and relative 95% confidence interval (CI). Univariate and multivariate analyses were performed with the Cox proportional model to determine the effects of independent prognostic factors on DFS and OS. Calculating the exponential of the regression coefficients from the Cox model provided an estimate of the HR and the 95% CI. DFS and OS curves were calculated using the Kaplan–Meier method and compared using the log-rank test. Two-tailed *p* < 0.05 were considered statistically significant.

## Results

### Patient Characteristics and Responses

We collected the information of 105 BC patients who received NAC before surgery (**Tables 1**, **2**). The median age was 45.4 years (range, 28–66 years). All patients were diagnosed with stage II or III disease, and 71.4% of them were premenopausal at diagnosis. The clinical response to NAC was assessed for each patient and summarized by the RECIST 1.1 criteria. The overall clinical PR rate was 66.7% (70/105), and the clinical SD rate was 30.5% (32/105), and the remainder of the patients had PD. The pathological responses were assessed by MP grades. Six patients (5.7%) had an MP grade 5 response, 9 patients (8.6%) had a grade 4 response, 40 patients (38.1%) had a grade 3 response, 36 patients (34.3%) had a grade 2 response, and 14 patients (13.3%) had a grade 1 response. The 105 patients were classified into two subtypes: Luminal-A (21.9%, n = 23), 1 patient (4.3%) was grade 4, 8 patients (34.8%) were grade 3, 8 patients (34.8%) were grade 2, and 6 patients (26.1%) were MP grade 1; Luminal-B (69.5%, n = 73), 5 patients (6.8%) were grade 5, 7 patients (9.6%) were grade 4, 30 patients (41.1%) were grade 3, 25 patients (34.2%) were grade 2, and 6 patients (8.2%) were MP grade 1. The overall pCR was 3.8% (4/105), considering both MP grades and residual nodes. Thirty-eight patients (36.2%) received adjuvant chemotherapy; 90 patients (85.7%) received adjuvant radiotherapy. Type of endocrine therapy used was tamoxifen in 33 (31.4%), aromatase inhibitor (AI) + ovarian function suppression in 47 (44.8%), and AI alone in 31 (29.5%). A total of 54 patients had available postoperative tissue for the 70-gene signature (**Supplementary Table S1**).

**Table 1:** The preoperative clinicopathological characteristics of patients.

Variable	Result n = 105 (n%)
Mean age, years (range)	45.4 (28–66)
Menopausal status	
Premenopausal	75 (71.4)
Postmenopausal	30 (28.6)
Tumor stage	
T1	10 (9.5)
T2	62 (59.0)
T3	17 (16.2)
T4	16 (15.2)
Nodal stage	
N0	10 (9.5)
N1	42 (40.0)
N2	26 (24.8)
N3	27 (25.7)
Clinical stage	
II	42 (40.0)
III	63 (60.0)
ER expression	
<60%	7 (6.7)
≥60%	91 (86.7)
Unknown	7 (6.7)
PgR expression	
≤20%	46 (43.8)
>20%	52 (49.5)
Unknown	7 (6.7)
KI67	
<14%	17 (16.2)
≥14%	79 (75.2)
Unknown	9 (8.6)
Subtype (based on receptor status)	
Luminal A	23 (21.9)
Luminal B	73 (69.5)
Unknown	9 (8.6)
Primary systemic therapy	
Anthracycline-based	17 (16.2)
Anthracycline-taxane	88 (83.8)
Clinical response	
PR	70 (66.7)
SD	32 (30.5)
PD	3 (2.9)

ER, estrogen receptor; PgR, progesterone receptor; SD, stable disease; PD, progressive disease; PR, partial response.

**Table 2:** The postoperative clinicopathological characteristics of patients.

Variable	Result n = 105 (n%)
ER expression	
<60%	5 (4.8)
≥60%	92 (87.6)
Unknown	8 (7.6)
PgR expression	
≤20%	50 (47.6)
>20%	47 (44.8)
Unknown	8 (7.6)
KI67	
<14%	43 (41.0)
≥14%	54 (51.4)
Unknown	8 (7.6)
Surgery breast	
Mastectomy	94 (89.5)
Breast conserving surgery	11 (10.5)
Lymphovascular invasion	
No	64 (61.0)
Yes	41 (39.0)
Nerve invasion	
No	81 (77.1)
Yes	24 (22.9)
Grade	
I	3 (2.9)
II	69 (65.7)
III	18 (17.1)
Unknown	15 (14.3)
Pathological reaction	
pCR	4 (3.8)
Non-pCR	101 (96.2)
Miller–Payne grades	
1	14 (13.3)
2	36 (34.3)
3	40 (38.1)
4	9 (8.6)
5	6 (5.7)
MammaPrint	
Low risk	26 (24.8)
High risk	28 (26.7)
Unknown	51 (48.6)

pCR, pathological complete response; AI, aromatase inhibitor; OFS, ovarian function suppression.

### Logistic Analysis of Pretreatment Factors and MP Grades

Univariate logistic regression analysis revealed that good response (Grades 3–5) to NAC in early-stage HR+/HER2− BC was significantly associated with N-stage 2–3, Anthracycline-taxane or clinical PR (N-stage: *p* = 0.042, OR 2.25, 95% CI 1.03–4.92; primary systemic therapy: *p* = 0.014, OR 4.48, 95% CI 1.35–14.84; clinical response: *p* = 0.010, OR 0.33, 95% CI 0.14–0.77), while it was unrelated to age, clinical tumor stage, clinical stage, PR, Ki-67, and molecular subtypes (**Table 3**).

**Table 3:** Logistic univariate and multivariate analyses for the relationship between clinicopathological characteristics pretreatment and Miller–Payne grades in 105 patients.

		Univariate			Multivariate		
		OR	95% CI	*p-*value	OR	95% CI	*p-*value
Age at diagnosed (years)							
<50		1.00					
≥50		1.33	(0.59–3.02)	0.490			
Tumor stage							
T1–T2		1.00					
T3–T4		1.14	(0.50–2.59)	0.764			
Nodal stage							
N0–N1		1.00					
N2–N3		2.25	(1.03–4.92)	0.042			
Clinical stage							
II		1.00					
III		1.90	(0.86–4.18)	0.112			
PR expression							
≤20%		1.00					
>20%		0.60	(0.27–1.34)	0.216			
KI67							
<14%		1.00					
≥14%		2.43	(0.82–7.22)	0.111			
Subtype (based on receptor status)							
Luminal A		1.00					
Luminal B		2.11	(0.81–5.49)	0.127			
Primary systemic therapy							
Anthracycline-based		1.00			1.00		
Anthracycline-taxane		4.48	(1.35–14.84)	0.014	4.35	(1.20–15.76)	0.025
Clinical response							
PR		1.00			1.00		
SD-PD		0.33	(0.14–0.77)	0.010	0.34	(0.13–0.87)	0.024

OR, odds ratio; CI, confidence interval.

Multivariate analysis was performed using baseline characteristics with *p* < 0.15 in the univariate analysis. The results showed that good response to NAC in early-stage HR+/HER2− BC was significantly associated with anthracycline-taxane chemotherapy or clinical PR (*p* < 0.05). The other factors were not related to good response. ER could not be analyzed for MP grades because most patients had tumors with high ER expression.

### Characteristics of the Postoperative Tumor in the MammaPrint Cohort

**Figure 2:**
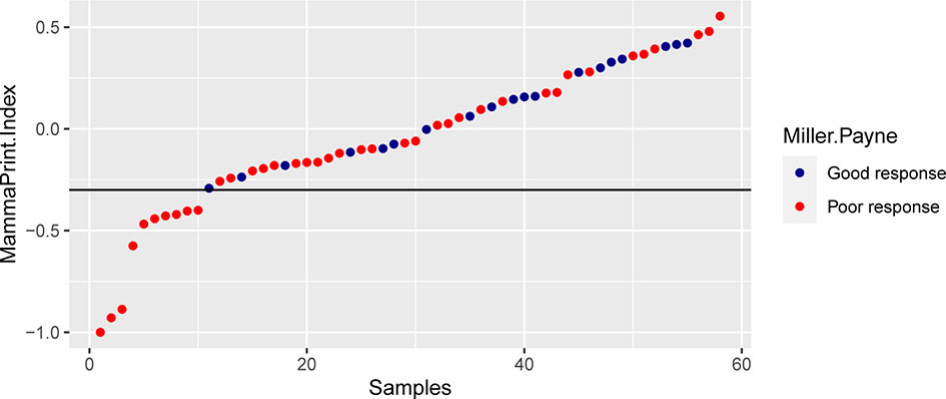
The association between the classification of the 70-gene recurrence risk and the pathological response.

The 70-gene signature was analyzed in 54 patients; the tumor characteristics are presented in **Supplementary Table S1** and **Table 4**. The 54 patients were classified into two subtypes: Luminal-A (18.5%, n = 10), 6 patients (60.0%) had low- and 4 patients (40.0%) had high-risk signature; Luminal-B (74.1%, n = 40), 18 patients (45.0%) had low- and 22 patients (55.0%) had high-risk signature. Among the 54 early HR+/HER2− patients, 26 (48.1%) had a low-risk signature, whereas 28 (51.9%) patients had a high-risk signature. Tumors with a high-risk signature were of higher grade, higher Ki67 level, and were more often classified as Luminal B tumors. **Figure 2** shows the relation between the classification of the 70-gene profile as a continuous variable and MP grades (good response: grades 3–4; poor response: grades 1–2). Patients with a good response have a higher probability to with a high MammaPrint Index.

**Table 4:** The tumor characteristics of postoperative and the association with the 70-gene signature.

Variables	Overall	High risk	Low risk	*p*-value
	n = 54 (%)	n = 28 (51.9%)	n = 26 (48.1%)	
Grade				0.001
I	2 (3.7)	0 (0)	2 (7.7)	
II	36 (66.7)	18 (64.3)	18 (69.2)	
III	11 (20.4)	10 (35.7)	1 (3.8)	
unknown	5 (9.3)	0 (0)	5 (19.2)	
PR expression				0.172
≤20%	29 (53.7)	18 (64.3)	11 (42.3)	
>20%	25 (46.3)	10 (35.7)	15 (57.7)	
KI67				0.000
<14%	21 (38.9)	4 (14.3)	17 (65.4)	
≥14%	33 (61.1)	24 (85.7)	9 (34.6)	
Subtype (based on receptor status)				0.006
Luminal A	15 (27.8)	3 (10.7)	12 (46.2)	
Luminal B	39 (72.2)	25 (89.3)	14 (53.8)	
Tumor stage				0.916
T1	21 (38.9)	12 (42.9)	9 (34.6)	
T2	27 (50.0)	13 (46.4)	14 (53.8)	
T3	4 (7.4)	2 (7.1)	2 (7.8)	
T4	2 (3.7)	1(3.6)	1 (3.8)	
Nodal stage				0.490
N0	14 (25.9)	6 (21.4)	8 (30.8)	
N1	18 (33.3)	9 (32.1)	9 (34.6)	
N2	11 (21.4)	5 (17.9)	6 (23.1)	
N3	11 (21.4)	8 (28.6)	3 (11.5)	
Stage				0.748
I	7 (13.0)	4 (14.3)	3 (11.5)	
II	24 (44.4)	11 (39.3)	13 (50.0)	
III	23 (42.6)	13 (46.4)	10 (38.5)	
Miller–Payne grade				0.155
½	35 (64.8)	21 (75.0)	14 (53.8)	
¾	19 (35.2)	7 (25.0)	12 (46.2)	

### Survival Analysis

Of the 105 early-stage HR+/HER2− cases, the magnitude of impact on different prognosis including DFS and OS differed depending on factors (**Supplementary Table S2**). Multivariate analysis showed that larger tumor size and lower PR level at baseline and larger tumor size postoperative were independent predictor of DFS.

**Figure 3:**
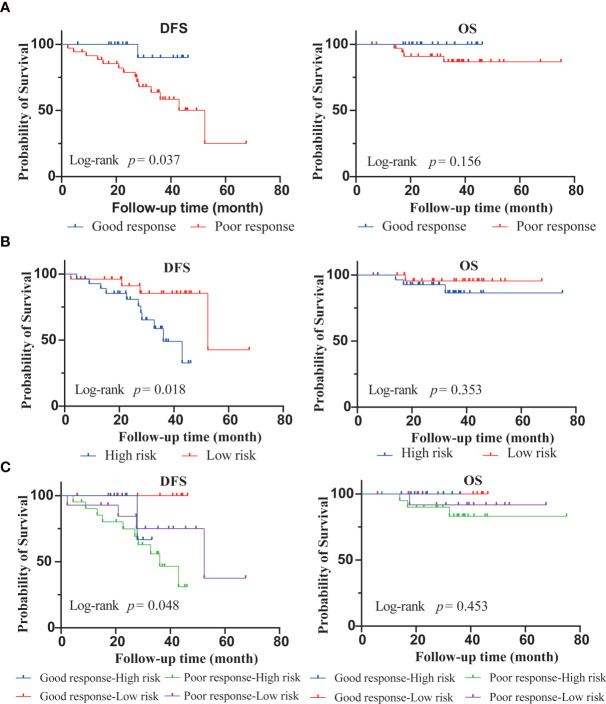
Disease-free survival (DFS) and overall survival (OS) according to Miller–Payne grades **(A)** and MammaPrint evaluation **(B)**. Kaplan-Meier DFS and OS estimates for combinations of Miller–Payne grades and MammaPrint evaluation **(C)**.

Among 54 patients who underwent the 70-gene assays, there were four incidences of death. The median follow-up time was 34.5 months (5.9–75.1 months). Postoperative clinicopathological factors were analyzed for survival (**Table 5**). Univariate analysis showed that DFS was significantly associated with KI67, pathological tumor stage, and MammaPrint signature. OS was related to the pathological tumor stage (*p* < 0.05); however, it was unrelated to histological grade, PR, KI67, molecular subtypes, nodal stage, pathological stage, MP grades, and MammaPrint signature (**Figure 3**). Multivariate analysis was performed using postoperative characteristics with *p* < 0.15 in the univariate analysis. The results showed that pathological tumor stage was independent predictor of DFS and OS in early-stage HR+/HER2− BC after NAC.

**Table 5:** Univariate and multivariate analysis of postoperative factors predictive of disease-free survival and overall survival in MammaPrint group.

(Univariate analysis)							
Variable	Disease-free survival				Overall survival		
	HR	95% CI	*p*-value		HR	95% CI	*p*-value
Grade							
G1–G2	1.00				1.00		
G3	1.17	(0.32–4.29)	0.808		3.79	(0.53–26.93)	0.183
PR expression							
≤20%	1.00				1.00		
>20%	0.92	(0.33–2.53)	0.866		0.02	(0.00–54.73)	0.324
KI67							
<14%	1.00				1.00		
≥14%	4.92	(1.09–22.19)	0.038		1.83	(0.19–17.57)	0.602
Subtype (based on receptor status)							
Luminal A	1.00				1.00		
Luminal B	2.26	(0.50–10.15)	0.287		32.68	–	0.469
Tumor stage			0.000				0.047
T1	1.00				1.00		
T2	0.45	(0.13–1.55)	0.207		0.52	(0.03–8.50)	0.645
T3–T4	10.43	(2.29–47.59)	0.002		8.74	(0.78–97.41)	0.078
Nodal stage							
N0–N1	1.00				1.00		
N2–N3	2.00	(0.71–5.62)	0.191		1.38	(0.19–9.78)	0.749
Stage							
I–II	1.00				1.00		
III	1.98	(0.70–5.56)	0.197		1.38	(0.19–9.78)	0.749
Miller–Payne grades							
1/2	1.00				1.00		
3/4	6.53	(0.85–49.99)	0.071		38.97	–	0.410
MammaPrint signature							
Low Risk	1.00				1.00		
High Risk	0.24	(0.07–0.87)	0.029		0.36	(0.04–3.44)	0.373
(Multivariate analysis)							
Variable	Disease-free survival				Overall survival		
	HR	95% CI	*p*-value		HR	95% CI	*p* value
Tumor stage			0.001				0.047
T1	1.00				1.00	
T2	0.18	(0.04–0.83)	0.027		0.52	(0.03–8.50)	0.645
T3–T4	5.91	(1.32–26.43)	0.020		8.74	(0.78–97.41)	0.078

The study showed that DFS was longer in the good response (MP grades 3–4) group than in the poor response (MP grades 1–2) group (94.7% vs. 60% of the patients free from recurrence; HR, 0.16; 95% CI, 0.05–0.47; *p* = 0.037) (**Figure 3A**). DFS was longer in the low-risk group than in the high-risk group (52.4 vs. 36.1 months of the median DFS; HR for recurrence, 0.29; 95% CI, 0.10–0.80; *p* = 0.018) (**Figure 3B**). When stratified by MP grades and the 70-gene signature, subgroup analyses showed the -response low-risk group with the best DFS, whereas the poor-response high-risk group showed the worst DFS (*p* = 0.048, **Figure 3C**).

## Discussion

The pCR rate is associated with tumor subtypes; the HER2+ and triple-negative subtypes had higher pCR rates than the HR+/HER2− subtype (3, 19). In this study, a series of 105 patients with stage II–III primary invasive HR+/HER2− BC who received NAC were described. The overall pCR was 3.8% (4/105), which was consistent with previous studies. Therefore, pCR cannot be used to evaluate pathological responses to HR+/HER2− BC after NAC, and more accurate prediction alternative markers are needed. The overall loss of cellularity after NAC is not always reflected by a decrease in tumor size, as the fibrous stroma still exists. The MP grading system is based on the degree of tumor cell loss, which occurs within the tumor during NAC. Therefore, MP grades can accurately evaluate the main manifestation of pathological response to NAC and identify patients with a better prognosis (11, 20). Our study showed that only six patients (5.7%) had a MP grade 5 response, many patients exhibiting residual tumor cells were present, and the reaction to NAC may correlated with survival benefit. Multivariate analysis showed that anthracycline-taxane chemotherapy or clinical response was significantly associated with good response (MP grades 3–5). Therefore, anthracycline-taxane regimen was a better choice for HR+/HER2− BC who received NAC.

This study demonstrated that larger tumor size and lower progesterone receptor level at baseline and larger tumor size of postoperative were statistically significantly associated with worse DFS (*p* = 0.004, *p* = 0.021 and *p* = 0.001, respectively). However, a previous study showed that lower estrogen receptor level after 2 weeks of endocrine therapy is associated with poorer recurrence-free survival (21). In our study, ER could not be analyzed as most patients had tumors with high ER expression. A possible explanation for this phenomenon may be that significant changes in tumor hormone receptors status occur following NAC (22).

Patients with residual BC after NAC are at a high risk of recurrence of metastases, which make these patients ideal applicants for prognostic analysis. Residual cancer burden (RCB) score can be as a prognostic marker for long-term survival after NAC independent of the molecular subtype (23). The prognostic value of posttreatment Oncotype DX Recurrence Score (RS) results in tumor specimens from patients receiving neoadjuvant endocrine therapy (NET) was evaluated in a study by Ueno et al. (24), which demonstrated that posttreatment RSs was significantly associated with DFS. Next-generation sequencing-based multigene assay have been validated to have prognostic in early-stage HR+/HER2− BC (8). A study showed that neoadjuvant therapy can significantly altered MammaPrint index and molecular subtype, indicating that the genetic characteristics of the residual tumor are different from pretreatment genomic profile (25). To better delineate the relapse risk in patients after NAC with residual disease, it is necessary to develop a multigene assay to predict prognosis.

The residual chemotherapy-resistant cancer cells in the breast and nodes after NAC may harbor different gene expression patterns and DNA mutational signatures, which would result in differential survival. In this study, 54 resection breast samples met the 70-gene profile quality requirements and performed genomic assays. The proportion of tumors with a low-risk signature was 48.1%; that of high-risk signature was 51.9%. We observed that tumors with poor response (MP Grades 1–2) were more likely to have a high-risk 70-gene signature than those with good response (MP Grades 4–5) (**Figure 1**). Our study showed that MP grades and the 70-gene signature were statistically significantly associated with DFS (*p* = 0.037, *p* = 0.018, respectively). Stratifying by MP grades and the 70-gene signature also showed marked prognostic value (*p* = 0.048). The poor-response high-risk group showed the worst DFS, whereas the good-response low-risk group showed the best DFS. All patients received tamoxifen or aromatase inhibitors combined with ovarian function suppression adjuvant endocrine therapy. The study showed that active endocrine intervention for the poor-response high-risk group was not enough; the addition of chemotherapy or intensive endocrine therapy (e.g., cyclin-dependent kinase 4/6 inhibitors, CDK4/6i) in the adjuvant setting may prolong DFS and improve prognosis for these patients. Due to the short follow-up time, MP grades and the 70-gene signature did not show significant prognostic value for OS. A previous study used tumor samples prior to NAC for the 70-gene, which signature showed good prognostic value and predictive chemotherapeutic sensitivity (26). However, this study used residual chemotherapy-resistant breast samples, which showed similar prognostic value. This finding has important clinical implication. Based on the prognostic value of the 70-gene signature in HR+/HER2− early-stage BC who have residual invasive carcinoma after NCA; further clinical studies of intensive adjuvant therapy in high-risk patients should be conducted.

There were several limitations that should be considered while interpreting the study findings. One critical limitation was the small sample size that restricted our analysis for the subgroup based on MP grades and the 70-gene signature. Another limitation was that a longer follow-up will be required to confirm that MP grades and the 70-gene signature were associated with long-term survival. Different NAC regimens and cycles were another issue. In the present study, most patients received different cycles of anthracycline-based or anthracycline-taxane NAC, which may affect a reduction in tumor load of the primary tumor. Despite these limitations, our study has several strengths. To our knowledge, this is the first study that provides an analysis of MP grades combined with the 70-gene signature associated with prognostic in HR+/HER2− non-pCR populations. Integration of MP grades and the 70-gene signature to determine the appropriate options of adjuvant treatment may prolong DFS and improve prognosis. Further studies are warranted to confirm the clinical utility of MP grades combined with the 70-gene signature in early-stage HR+/HER2− breast cancer.

In conclusion, this study showed that analysis of MP grades combined with the 70-gene signature with residual neoadjuvant chemotherapy-resistant breast samples has a significant correlation with DFS. In particular, subgroup analyses based on MP grades and the 70-gene signature also showed markedly prognostic value.

## Data Availability Statement

The original contributions presented in the study are included in the article/**Supplementary Material**. Further inquiries can be directed to the corresponding authors.

## Author Contributions

Conception and design of the study: FX and SW. Acquisition of clinical data: LW, QL, KJ, RH, KL, PZ, and DZ. Miller–Payne grades pathological evaluation: RL. Analysis and interpretation of the data: LW and QL. Manuscript drafting and revision: LW, QL, FX, and SW. All authors contributed to the article and approved the submitted version.

## Funding

The work was funded by the Sun Yat-sen University Clinical Research 5010 Program (2017010).

## Conflict of Interest

The authors declare that the research was conducted in the absence of any commercial or financial relationships that could be construed as a potential conflict of interest.

## Publisher’s Note

All claims expressed in this article are solely those of the authors and do not necessarily represent those of their affiliated organizations, or those of the publisher, the editors and the reviewers. Any product that may be evaluated in this article, or claim that may be made by its manufacturer, is not guaranteed or endorsed by the publisher.
