# Supplementary material for: Miller–Payne Grading and 70-Gene Signature Are Associated With Prognosis of Hormone Receptor-Positive, Human Epidermal Growth Factor Receptor 2-Negative Early-Stage Breast Cancer After Neoadjuvant Chemotherapy
Source: Front Oncol. 2021 Sep 24;11:735670. doi: 10.3389/fonc.2021.735670 (PMC8498026; doi:10.3389/fonc.2021.735670)
Supplement: Supplementary file 1 [file Table_1.docx]

Table 1 The clinicopathological characteristics of MammaPrint group patients.

| Variable | Result n=54 (n%) | |
| --- | --- | --- |
|  | preoperative | postoperative |
| Mean age, years (range) | 46.7 (32-65) | 46.7 (32-65) |
| Menopausal status |  |  |
| Pre-menopausal | 38 (70.4) | 38 (70.4) |
| Post-menopausal | 16 (29.6) | 16 (29.6) |
| Tumor stage |  |  |
| T1 | 3 (5.6) | 21 (38.9) |
| T2 | 32 (59.3) | 27 (50.0) |
| T3 | 9 (16.7) | 4 (7.4) |
| T4 | 10 (18.5) | 2 (3.7) |
| Nodal stage |  |  |
| N0 | 9 (16.7) | 14 (25.9) |
| N1 | 20 (37.0) | 18 (33.3) |
| N2 | 11 (20.4) | 11 (20.4) |
| N3  stage  I  II  III  ER expression  < 60%  ≥ 60%  PgR expression | 14 (25.9)  0  22 (20.7)  32 (59.3)  1 (1.9)  53 (98.1) | 11 (20.4)  7 (13.0)  24 (44.4)  23 (42.6)  2 (3.7)  52 (96.3) |
| ≤20% | 22 (40.7) | 29 (53.7) |
| >20% | 32 (59.3) | 25 (46.3) |
| KI67 |  |  |
| <14% | 10 (18.5) | 21 (38.9) |
| ≥14%  unknown | 40 (74.1)  4 (7.4) | 33 (61.1)  0 |
| Subtype (based on receptor status) |  |  |
| Luminal A | 10 (18.5) | 15 (27.8) |
| Luminal B  unknown | 40 (74.1)  4 (7.4) | 39 (72.2)  0 |
| Primary systemic therapy |  |  |
| Anthracycline-based | 11 (20.4) |  |
| Anthracycline-taxane | 43 (79.6) |  |
| Clinical response  PR  SD | 33 (61.1)  21 (38.9) |  |
| Surgery breast |  |  |
| Mastectomy |  | 49 (90.7) |
| Breast conserving surgery |  | 5 (9.3) |
| Lymphovascular invasion |  |  |
| No |  | 29 (53.7) |
| Yes |  | 25 (46.3) |
| Nerve invasion |  |  |
| No |  | 37 (68.5) |
| Yes |  | 17 (31.5) |
| Grade |  |  |
| I |  | 2 (3.7) |
| II |  | 36 (66.7) |
| III |  | 11 (20.4) |
| unknown |  | 5 (9.3) |
| Miller-Payne grades |  |  |
| 1 |  | 12 (22.2) |
| 2 |  | 23 (42.6) |
| 3 |  | 18 (33.3) |
| 4 |  | 1 (1.9) |
| Postoperative treatment |  |  |
| Chemotherapy |  | 54 (100.0) |
| Endocrine therapy |  | 54 (100.0) |
| Radiotherapy |  | 47 (87.0) |
| Endocrine therapy |  |  |
| Tamoxifen |  | 10 (18.5) |
| AI |  | 19 (35.2) |
| AI+OFS |  | 25 (46.3) |
| MammaPrint |  |  |
| Low Risk |  | 26 (48.1) |
| High Risk |  | 28 (51.9) |

ER: Estrogen receptor; PgR: Progesterone receptor; PR: Partial response; SD: Stable disease; AI: Aromatase inhibitor; OFS: Ovarian function suppression.
