# Supplementary material for: Miller–Payne Grading and 70-Gene Signature Are Associated With Prognosis of Hormone Receptor-Positive, Human Epidermal Growth Factor Receptor 2-Negative Early-Stage Breast Cancer After Neoadjuvant Chemotherapy
Source: Front Oncol. 2021 Sep 24;11:735670. doi: 10.3389/fonc.2021.735670 (PMC8498026; doi:10.3389/fonc.2021.735670)
Supplement: Supplementary file 2 [file Table_2.docx]

Table 2 Univariate and multivariate analysis of factors predictive of disease-free survival and overall survival in all patients.

(Univariate analysis)

| Variable | Disease-free survival | | | Overall survival | | |
| --- | --- | --- | --- | --- | --- | --- |
|  | HR | 95 % CI | *p* Value | HR | 95 % CI | *p* Value |
| Pretreatment factors | | | | | | |
| Age at diagnosed (years) | | |  |  |  |  |
| ＜50 | 1.00 |  |  | 1.00 |  |  |
| ≥ 50 | 1.14 | (0.56-2.33) | 0.720 | 1.12 | (0.33-3.84) | 0.854 |
| Tumor stage | | |  |  |  |  |
| T1-T2 | 1.00 |  |  | 1.00 |  |  |
| T3-T4 | 2.55 | (1.27-5.11) | 0.008 | 1.19 | (0.35-4.08) | 0.779 |
| Nodal stage | | |  |  |  |  |
| N0-N1 | 1.00 |  |  | 1.00 |  |  |
| N2-N3 | 2.59 | (1.24-5.39) | 0.011 | 5.83 | (1.24-27.36) | 0.025 |
| Clinical stage | | |  |  |  |  |
| II | 1.00 |  |  | 1.00 |  |  |
| III | 2.51 | (1.12-5.60) | 0.025 | 3.48 | (0.75-16.21) | 0.112 |
| PR expression | | |  |  |  |  |
| ≤ 20% | 1.00 |  |  | 1.00 |  |  |
| ＞20% | 0.28 | (0.13-0.65) | 0.003 | 0.014 | (0.00-2.24) | 0.099 |
| KI67 | | |  |  |  |  |
| < 14% | 1.00 |  |  | 1.00 |  |  |
| ≥ 14% | 3.89 | (0.91-16.58) | 0.067 | 2.58 | (0.32-20.55) | 0.371 |
| Subtype (based on receptor status) | | | |  |  |  |
| Luminal A | 1.00 |  |  | 1.00 |  |  |
| Luminal B | 5.97 | (1.39-25.67) | 0.016 | 36.91 | - | 0.225 |
| Postoperative factors | | | | | | |
| PR expression | | |  |  |  |  |
| ≤ 20% | 1.00 |  |  | 1.00 |  |  |
| > 20% | 0.73 | (0.35-1.54) | 0.412 | 0.14 | (0.02-1.09) | 0.060 |
| KI67 | | |  |  |  |  |
| < 14% | 1.00 |  |  | 1.00 |  |  |
| ≥ 14% | 1.80 | (0.84-3.88) | 0.134 | 1.22 | (0.32-4.61) | 0.767 |
| Subtype (based on receptor status) | | | |  |  |  |
| Luminal A | 1.00 |  |  | 1.00 |  |  |
| Luminal B | 3.04 | (1.06-8.76) | 0.040 | 3.97 | (0.49-31.96) | 0.195 |
| Tumor stage | | |  |  |  |  |
| T0-T1 | 1.00 |  |  | 1.00 |  |  |
| T2 | 0.92 | (0.42-2.01) | 0.836 | 1.19 | (0.31-4.52) | 0.801 |
| T3-T4 | 8.46 | (2.84-25.25) | 0.000 | 4.63 | (0.89-24.05) | 0.068 |
| Nodal stage | | |  |  |  |  |
| N0-N1 | 1.00 |  |  | 1.00 |  |  |
| N2-N3 | 2.59 | (1.29-5.22) | 0.008 | 1.91 | (0.58-6.28) | 0.289 |
| Miller-Payne grade | |  |  |  |  |  |
| 1/2 | 1.00 |  |  | 1.00 |  |  |
| 3/4/5 | 1.78 | (0.88-3.60) | 0.111 | 2.01 | (0.59-6.89) | 0.265 |

(Multivariate analysis)

| Variable | Disease-free survival | | *p* Value |
| --- | --- | --- | --- |
|  | HR | 95 % CI |  |
| Pretreatment factors |  |  |  |
| Tumor stage |  |  |  |
| T1-T2 | 1.00 |  |  |
| T3-T4 | 4.52 | (1.62-12.61) | 0.004 |
| PR expression |  |  |  |
| ≤ 20% | 1.00 |  |  |
| ＞20% | 0.33 | (0.13-0.85) | 0.021 |
| Postoperative factors |  |  |  |
| Tumor stage |  |  | 0.001 |
| T0-T1 | 1.00 |  |  |
| T2 | 0.63 | (0.24-1.62) | 0.333 |
| T3-T4 | 6.03 | (1.63-22.27) | 0.007 |

PR: Progesterone receptor; HR: Hazard ratio; CI: Confidence interval.
